# A timescale for placental mammal diversification based on Bayesian modeling of the fossil record

**DOI:** 10.1016/j.cub.2023.06.016

**Published:** 2023-06-27

**Authors:** Emily Carlisle, Christine M. Janis, Davide Pisani, Philip C.J. Donoghue, Daniele Silvestro

**Affiliations:** 1 Bristol Palaeobiology Group, School of Earth Sciences, University of Bristol, Life Sciences Building, Tyndall Avenue, Bristol BS8 1TQ, UK; 2 Department of Biology, University of Fribourg, 1700 Fribourg, Switzerland; 3 Swiss Institute of Bioinformatics, 1700 Fribourg, Switzerland; 4 Department of Biological and Environmental Sciences, University of Gothenburg, 413 19 Gothenburg, Sweden; 5 Gothenburg Global Biodiversity Centre, 413 19 Gothenburg, Sweden; 6 Bristol Palaeobiology Group, School of Biological Sciences, University of Bristol, Life Sciences Building, Tyndall Avenue, Bristol BS8 1TQ, UK

## Abstract

The timing of the placental mammal radiation has been the focus of debate over the efficacy of competing methods for establishing evolutionary timescales. Molecular clock analyses estimate that placental mammals originated before the Cretaceous-Paleogene (K-Pg) mass extinction, anywhere from the Late Cretaceous to the Jurassic. However, the absence of definitive fossils of placentals before the K-Pg boundary is compatible with a post-Cretaceous origin. Nevertheless, lineage divergence must occur before it can be manifest phenotypically in descendent lineages. This, combined with the non-uniformity of the rock and fossil records, requires the fossil record to be interpreted rather than read literally. To achieve this, we introduce an extended Bayesian Brownian bridge model that estimates the age of origination and, where applicable, extinction through a probabilistic interpretation of the fossil record. The model estimates the origination of placentals in the Late Cretaceous, with ordinal crown groups originating at or after the K-Pg boundary. The results reduce the plausible interval for placental mammal origination to the younger range of molecular clock estimates. Our findings support both the Long Fuse and Soft Explosive models of placental mammal diversification, indicating that the placentals originated shortly prior to the K-Pg mass extinction. The origination of many modern mammal lineages overlapped with and followed the K-Pg mass extinction.

## Introduction

**Figure 1:**
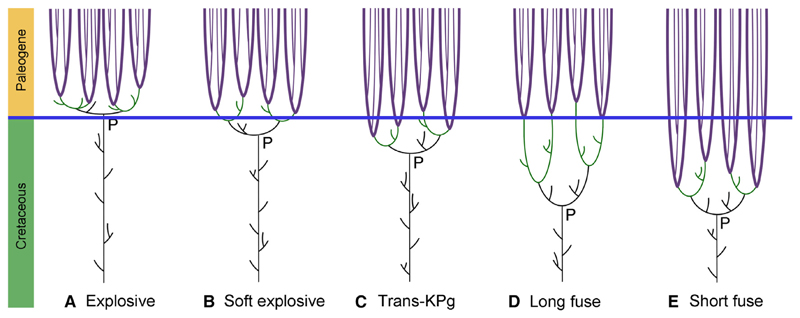
Patterns of placental mammal diversification Thick purple lines are crown orders, green lines are stem orders, and black lines are stem placental families. (A) Explosive model: all placental mammal diversification and origination occurred just after the K-Pg boundary. (B) Soft explosive model: placental mammals originated just before the K-Pg boundary, but intraordinal diversification only occurred after the boundary. (C) Trans-KPg model: both interordinal and intraordinal diversification occurred around the K-Pg boundary. (D) Long fuse model: placental mammals originated in the middle of Late Cretaceous, but intraordinal diversification did not begin until after the K-Pg boundary. (E) Short fuse model: placental origination and crown order diversification occurred during the Cretaceous.

The timing of the origin of placental mammals (crown eutherian mammals) has been the subject of a heated debate over how best to establish a timescale for evolutionary history. Several hypotheses have been proposed to explain the pattern of mammalian evolution around the Cretaceous-Paleogene (K-Pg) boundary, each supported by different studies. The Explosive Model (Figure 1A) of placental mammal evolution suggests that most or all of the placental radiation occurred after the K-Pg mass extinction^1–3^ based largely on a literal reading of the fossil record, from which no definitive placental mammal fossils have been found before the K-Pg boundary.^4,5^ However, the fossil record cannot be read literally, as the first stratigraphic appearances of taxa do not equate to their time of origination.^6,7^ The use of fossils as calibrations in molecular clock analyses allows for some interpretation of the fossil record, and the diversity of molecular clock methods has led to different hypotheses for the timing and tempo of the diversification of placental mammals.^2^ Yet, molecular clock studies consistently estimate a pre-Paleogene origin of Placentalia.^1,8–11^ Early strict molecular clock studies supported a Short Fuse Model (Figure 1E) in which both Placentalia and the placental orders are envisaged to have originated deep in the Cretaceous.^1,12^ This model lost favor with the application of relaxed molecular clock methods, which have supported a Long Fuse Model (Figure 1D) for placental mammal evolution. This proposes a Late Cretaceous origin of Placentalia but ordinal level crown groups not originating until after the K-Pg boundary.^1–3,8–10^ A variation of the Long Fuse Model is the Soft Explosive Model (Figure 1B), which posits that placental and some interordinal divergences occurred in the Cretaceous, but most of the inter- and intraordinal origination took place after the K-Pg boundary.^1,2^ The related Trans-K-Pg Model (Figure 1C) envisages interordinal diversification continuing at a steady rate throughout the Late Cretaceous and into the early Paleogene rather than the rapid radiation suggested by the Soft Explosive Model.^1,2,13^

Part of the difficulty in establishing the timeline of placental evolutionary history stems from the uncertainties of mammal phylogeny. Although it is widely accepted that the placental clades Laurasiatheria and Euarchontoglires are united to form Boreoeutheria, there is still uncertainty in the branching order of the clades Xenarthra, Afrotheria, and Boreoeutheria, which impacts the estimate of the age of origin.^9,14^ In particular, recent molecular clock analyses have tested numerous tree hypotheses, each resulting in slightly different age estimates.^9,11^ The choice of fossil calibrations and their placement in the tree can also have a large impact on age estimates from molecular clock analyses, potentially leading to biases imposed before analyses are run.^15–18^ Early debate was also confused by differences in the way that molecular biologists and paleontologists defined clades, with paleontologists focusing on crown groups and their morphological synapomorphies, whereas molecular clock analyses estimate the earliest point of divergence between clades, which is before morphological synapomorphies were acquired.^19,20^ This leads to a gap between the molecular divergence age and the first appearance of the recognizable lineage in the fossil record.^21^ This difference may contribute to the discrepancy observed between age estimates from the fossil record and molecular data, especially if, as has been shown for placental mammals, early crown members are difficult to distinguish from stem members.^22,23^

There have been several recent attempts to interpret the fossil record. Stratigraphic confidence intervals make use of the stratigraphic range of lineages and have been used to estimate origin ages based on the fossil record.^24,25^ However, it has proven difficult to account for changes in preservation potential through time.^21,25,26^ Estimates of the rate of fossil preservation suggested that placental orders were unlikely to have originated much earlier than their fossil records indicate.^27^ However, this method was based on the standard birth-death model that may have unrealistic assumptions, especially for clades with poor fossil records or where representative sampling methods are used, in large part because there is insufficient fossil data to distinguish between the numerous diversification scenarios that can produce the same timetrees.^28,29^ Additionally, this method relies on an accurate parameterization of the model describing the fossil data.^30–32^ Many methods, including molecular clock analyses, also require accurate phylogenies of the clades analyzed, and, where there is phylogenetic variability, competing topologies are often examined to determine the impact of uncertainty on the age estimates.^11^

Here, we present a means of estimating the origin of placental clades and, where applicable, their time of extinction, without the use of phylogenies or molecular data, making full use of the fossil record. Our model uses a clade’s fossil record of diversity through time to estimate a Brownian bridge back to when the diversity of the clade was one, providing an estimate for the age of origin. This is an extension of the original Bayesian Brownian bridge (BBB) model^33^ that could only estimate the age of extant clades using extant diversity as one endpoint for the Brownian bridge. Since the model does not use phylogeny or molecular data, it is less susceptible to the uncertainties surrounding mammal relationships and to uneven sampling of mammal families. The inclusion of extinct families allows for a comprehensive estimate for the history of the clade.

## Results

**Figure 2:**
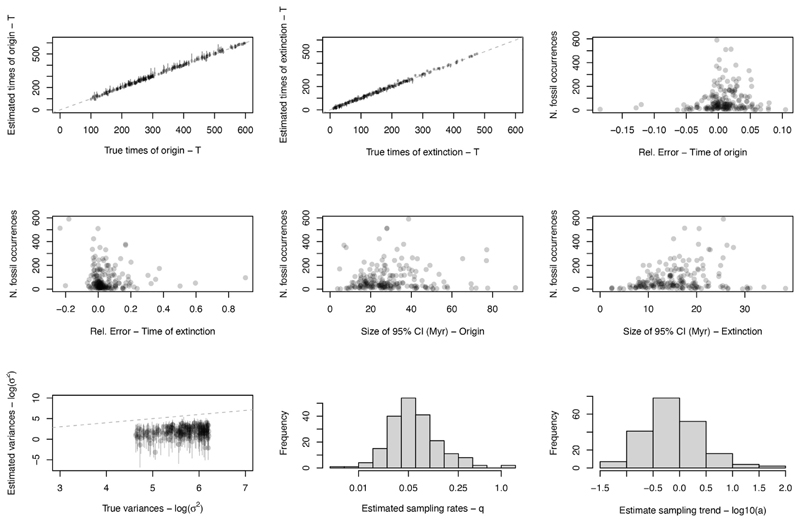
Simulations of 200 datasets of extinct clades The time of origin and time of extinction for each family are estimated accurately, and relative errors for both estimates are generally small, centered on zero and smaller for families with an abundant fossil record. Credible intervals were typically around 20 million years in size, with the size of the credible interval for extinction estimates tending to be smaller than that for the origin. The log variances were underestimated throughout, as observed in previous implementations of the model.^33^ Estimated sampling rates and sampling trends were generally small and unbiased.

**Figure 3:**
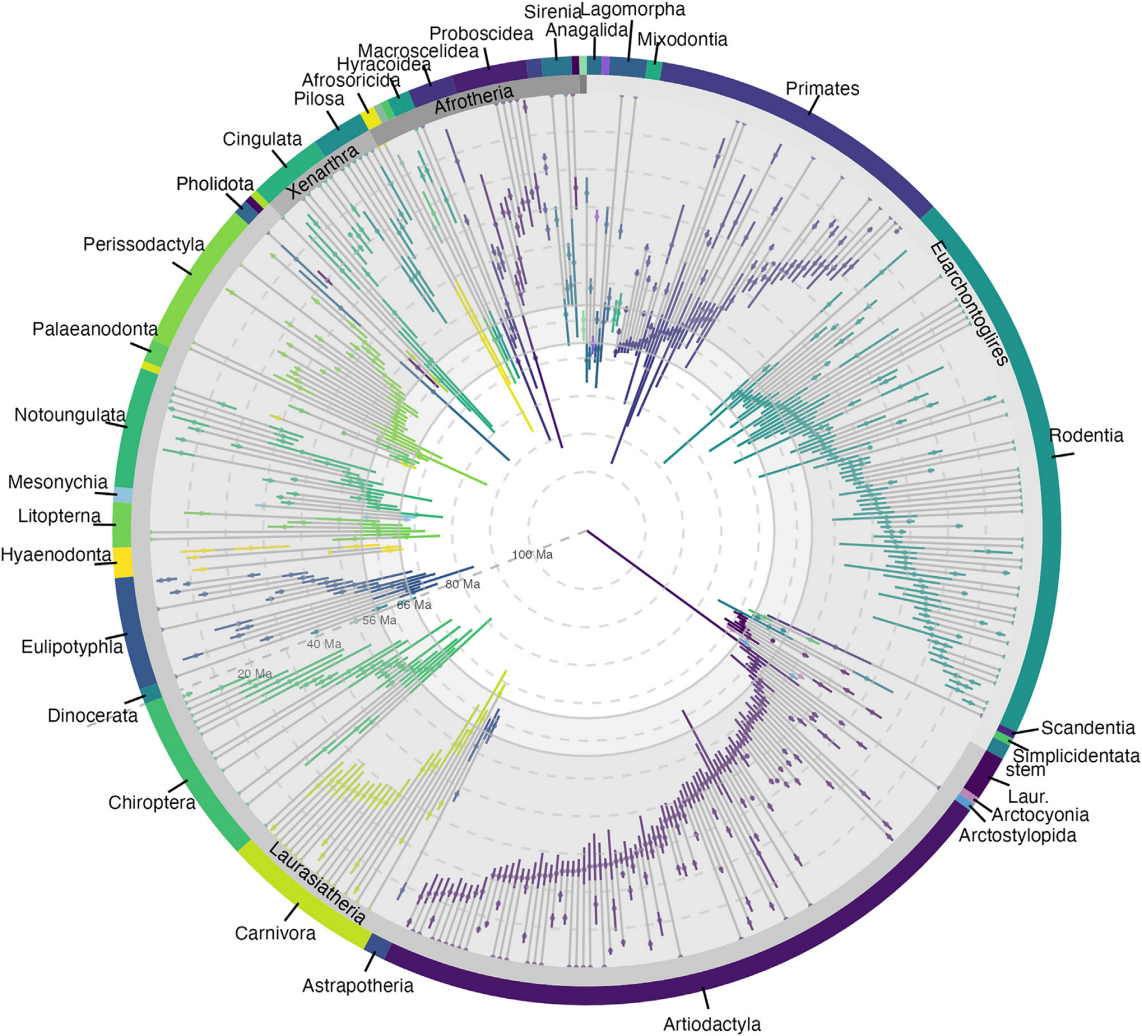
Clade age and extinction time estimates for placental mammal families Each line represents a family (arranged by order and clade but without further phylogenetic information), with 95% credible intervals in colors at the root estimates and extinction estimates (where applicable). Gray lines fill in the lineage. 93 families have credible intervals extending into the Cretaceous, but many originated after the K-Pg boundary. For stem and crown order classifications for each family, see Data S1.

Using the extended BBB model, we estimated the age for all mammalian families with a fossil record, extant and extinct. We first performed simulations to test the accuracy of the updated model when estimating both origination and extinction ages (STAR Methods). In these simulation tests, the estimated ages of origination and extinction were unbiased, and the size of the 95% credible intervals was typically between 10–20 million years (Myr), increasing in size with decreasing numbers of fossils (Figure 2). We then analyzed an empirical dataset of 380 placental mammal families. As expected, the size of the 95% credible interval is typically larger for families with fewer fossils. The estimated sampling rate at the time of origin for placental mammals (q) ranged from 3.67e^−4^ to 1.64 (median of 0.162; Figure S3), and the trend parameter (a—which describes the pace of increase of the sampling rate through time) ranged from 0.569 to 50.9 (median of 6.82; Figure S4), comparable with values used in our simulations. The age of clades can be indirectly estimated based on the oldest family within the clade,

which provides a minimum bound on the age of the clade.^33^ For placental mammals, 21.3% of the families have credible intervals that extend into the Cretaceous (Figure 3). Ten families have the full 95% credible interval within the Cretaceous, providing high support for a Cretaceous origin for placental mammals. These families are from the clades Euarchontoglires (Mimotonidae, Anagalidae, Pseudictopidae, and Purgatoriidae) and Laurasiatheria (Hyopsodontidae, Apheliscidae, Arctocyonidae, Louisinidae, Henricosborniidae, and Periptychidae). Purgatoriidae are stem primates (however, see Halliday et al.^5^) and are, therefore, members of crown Euarchontoglires.^34^ Henricosborniidae are early diverging notoungulates^35^; Louisinidae are stem perissodactyls^36^ (however, see Tabuce^37^ and Zack et al.^38^); and Arctocyonidae have been interpreted as sister to Carnivora^5^ or placed within Euungulata^39–41^; as such, all three families are members of crown Laurasiatheria.^5,35,36,42^ The age estimates for these families indicate that, out of the three major placental lineages, at least Boreoeutheria originated within the Cretaceous.

Although many placental mammal families have poor fossil records in the Paleobiology Database (PBDB), with 39.2% having ten or fewer individual fossils, our simulations indicate that the extended BBB model is still able to accurately estimate the ages of origin and extinction for poorly sampled clades (Figure S5), with relative errors for the time of origin centered on zero, thus indicating unbiased estimates. However, the size of the 95% credible interval is more variable and can be as high as 80 Myr, indicating that lower fossil numbers are linked with decreasing precision of the estimates. For the placental mammal data, the maximum size of the 95% credible interval for families with fewer than ten fossils is 56.8 Myr; however, the median is 6.7 Myr, suggesting that most clades yield relatively precise age estimates.

To account for the uncertainty around the age of each fossil occurrence in the BBB model, we used random ages drawn from the stratigraphic ranges provided by PBDB for each fossil occurrence. Although we removed occurrences with age ranges over 20 Myr, some still had a large age range, which may lead to different results. First, we assessed the distribution of age ranges within the dataset. Out of 27,323 occurrences (before the removal of duplicate species), 2,127 had an age range between 10 and 20 Myr, whereas 18,060 occurrences had an age range smaller than 5 Myr, suggesting that few families would be potentially impacted by the use of random ages. To test the robustness of our approach and quantify the effects of randomly resampling fossil ages on the oldest placental mammals, we re-estimated the ages of the oldest 75 families 35 times. Seven of the ten families that previously had the full 95% credible interval for the age of origin within the Cretaceous were found to have fully Cretaceous origin estimates in all the additional analyses. These families (Anagalidae, Apheliscidae, Arctocyonidae, Hyopsodontidae, Periptychidae, Pseudictopidae, and Purgatoriidae) include crown families from both Euarchontoglires (Purgatoriidae) and Laurasiatheria (Arctocyonidae), as well as stem members of these clades.

Species-level uncertainty in the data impacts the age estimates of families. To examine this, we tested fossils that have been suggested to be Placentalia present in the Cretaceous: *Deccanolestes, Altacreodus, Protungulatum, Paleoungulatum*, and *Baioconodon*.^43^ With *Deccanolestes* included in the family Adapisoriculidae,^44,45^ the root age is estimated to be 77–70 millions of years before present (Ma), whereas without *Deccanolestes*, this drops to 70.2–66 Ma. This suggests that Adapisoriculidae originated in the Cretaceous regardless of whether *Deccanolestes* is included in the family. The inclusion of *Altacreodus* within the family Cimolestidae,^46^ similarly, has no effect on the age estimate of this family, in large part because of other species (such as *Maelestes*) of a similar age to that of *Altacreodus. Paleoungulatum* has been interpreted as a member of the family Periptychidae,^47^ and its inclusion increases the age of this family from 68.8–67 to 72.6–70 Ma. The other “condylarths” *Protungulatum* and *Baioconodon* are possible members of the family Arctocyonidae, and their inclusion has little impact on the age estimate of this family, increasing it slightly from 70.9–68 to 72.8–70 Ma.

Adapisoriculidae and Cimolestidae also had family-level uncertainty; Adapisoriculidae has been interpreted as non-placental eutherians,^5^ euarchontans,^45,48^ or afrotherians,^45^ whereas Cimolestidae has been interpreted as either non-placental eutherians^5^ or laurasiatherians.^46^ The age of origin for Euarchontoglires is already within the Cretaceous based on the presence of Purgatoriidae, which was estimated to have a wholly Cretaceous origin; the inclusion or exclusion of Adapisoriculidae in this clade does not affect this conclusion. Afrotheria, however, was estimated to originate around the K-Pg boundary, but not fully within the Cretaceous (Figure 6); the inclusion of Adapisoriculidae means Afrotheria originated within the Cretaceous. The Cretaceous origin for Laurasiatheria is supported by three placental families (Henricosborniidae, Arctocyonidae, and Louisinidae); therefore, the inclusion or exclusion of Cimolestidae has no impact on the conclusion of a Cretaceous origin for this clade.

**Figure 4:**
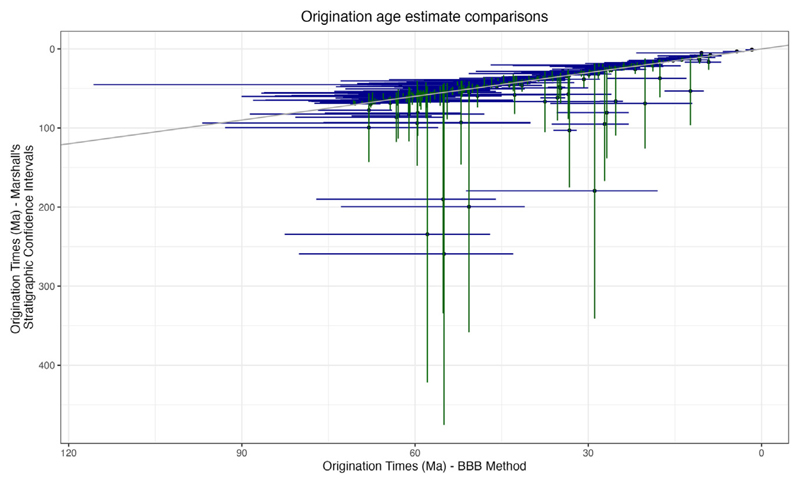
Clade age estimate comparison: BBB method and stratigraphic confidence intervals BBB estimates are shown in blue, and stratigraphic confidence intervals^25^ are shown in green. Although in many cases the estimates are similar, in some cases the stratigraphic method estimates very large confidence intervals. The slope of the line, which is for visual comparison, is one.

We also compared the results from the extended BBB model with confidence intervals calculated based on the stratigraphic record.^25^ The age of the oldest fossil informs the minimum of the confidence interval for the age of the family, whereas the maximum depends on the number of fossiliferous horizons and the size of the stratigraphic range during which the family is present. Although minima from both the BBB method and the stratigraphic method are similar, maxima vary substantially, reflecting the interplay between the number of fossiliferous horizons and the size of the stratigraphic range (Figure 4).

**Figure 5:**
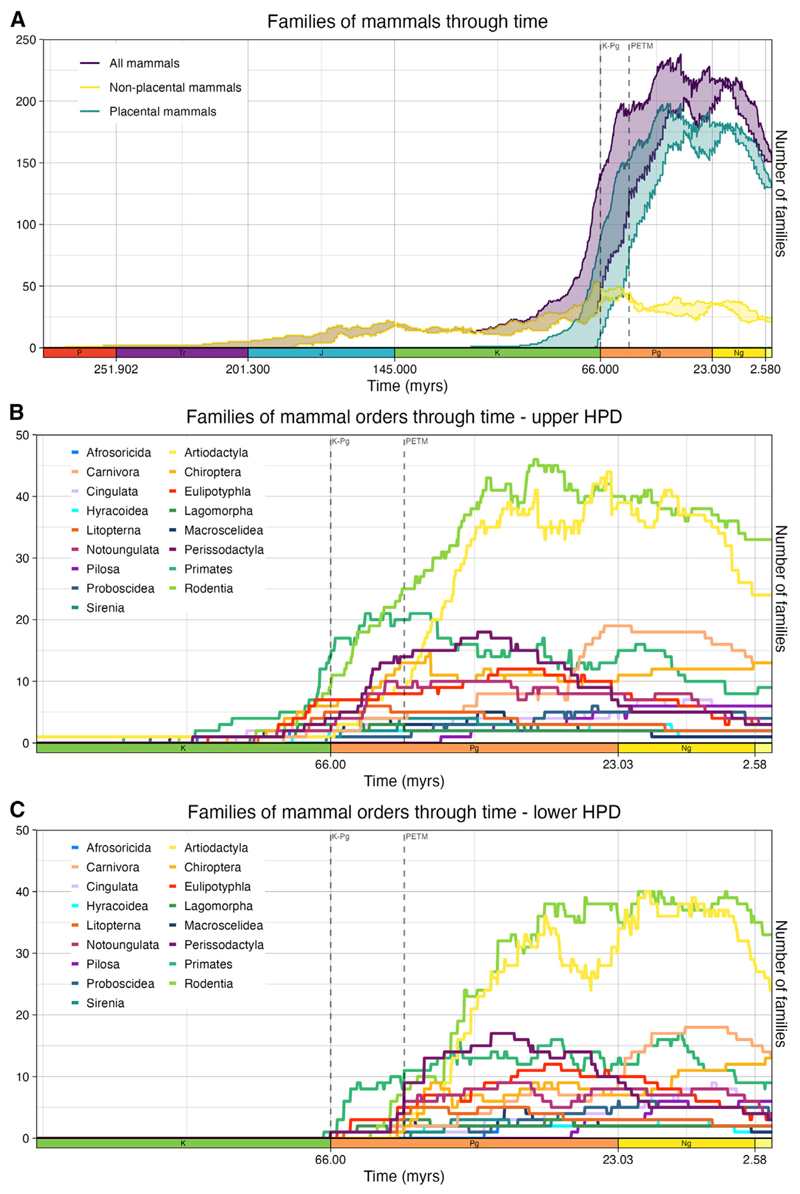
Lineages through time plot Lineages through time were calculated by adding and subtracting families based on their origination and extinction times (one line for the upper 95% highest posterior density (HPD) interval value, and one for the lower 95% HPD interval). (A) Lineages through the time of Mammalia, Placentalia, and non-placental mammal families. (B) Lineages through time based on the upper boundary of the credible interval for placental mammal orders. (C) Lineages through time based on the lower boundary of the credible interval for placental mammal orders. Note that stem and crown families were included in all orders.

The lineages through time plots demonstrate a rapid increase in placental mammal family richness in the 20 Myr following the K-Pg mass extinction, coinciding with the origin of several placental mammal orders (Figure 5). However, ordinal origination estimates are consistent with both a rapid post-K-Pg radiation of families and a Cretaceous origin for most of the orders (Figures 5B and 5C).

## Discussion

Simulation tests with the extended BBB model demonstrate that even for families with poor fossil records (i.e., ten or fewer individual fossils), the age of origin estimates were still accurate, with relative errors centered on zero. This demonstrates that clades with a small number of fossils can still be analyzed using the BBB method. Although using more inclusive taxonomic ranks such as orders would allow for greater fossil counts in lineages, it would also obscure the heterogeneity in lineage fossilization and sampling rates. Instead, we use families that allow us to control for lineage heterogeneity while still providing enough fossil evidence.

The age of the oldest family in each clade provides an indirect estimate for the age of the clade itself. We use the 95% credible interval as the age range rather than focusing on the mean age estimate; this allows for the inclusion of uncertainty into our estimates. Since seven placental mammal families consistently have the entire 95% credible interval within the Cretaceous, these provide strong support for a Cretaceous origin of Placentalia. These families each have a fossil representative from ~66 Ma; the presence of fossils from a diversity of lineages just after the K-Pg boundary effectively requires a prior history to explain their descent from a shared common ancestor. No definitive placental mammals are found in the Cretaceous; our sensitivity tests exploring the impact of putative Cretaceous placentals demonstrate that they have no material effect on the conclusions.

Although there is substantial correspondence between the results of the BBB model and the age estimates based on the stratigraphic confidence intervals,^25^ the age estimates based on the latter can be extremely precise (approaching zero width of the confidence interval) when the number of fossiliferous horizons is large or extremely broad (greater than 600 Myr wide) when the number of fossiliferous horizons is small (Figure 4). Using the BBB model, the credible intervals are much more consistently and realistically sized, with a maximum size of approximately 60 Myr. For instance, the oldest fossil in the family Palaeomastodontidae is from approximately 33 Myr, and there are only two fossiliferous horizons for this family. Using the stratigraphic confidence intervals method,^25^ the family is estimated to have originated 263.6–33 Ma, resulting in a 230 Myr 95% confidence interval. Using the BBB method, the 95% credible interval for the root age of the family is 36–33.3 Ma, only 4 Myr wide. Both methods base the minimum age on the age of the oldest fossil but estimate the maximum age considerably differently, with the BBB method better able to handle families with scarce fossil records. An additional benefit of this approach is that, beyond the 95% interval, the BBB method estimates a full posterior distribution for clade age, which could be used directly as a prior distribution to calibrate molecular clock analyses.

**Figure 6:**
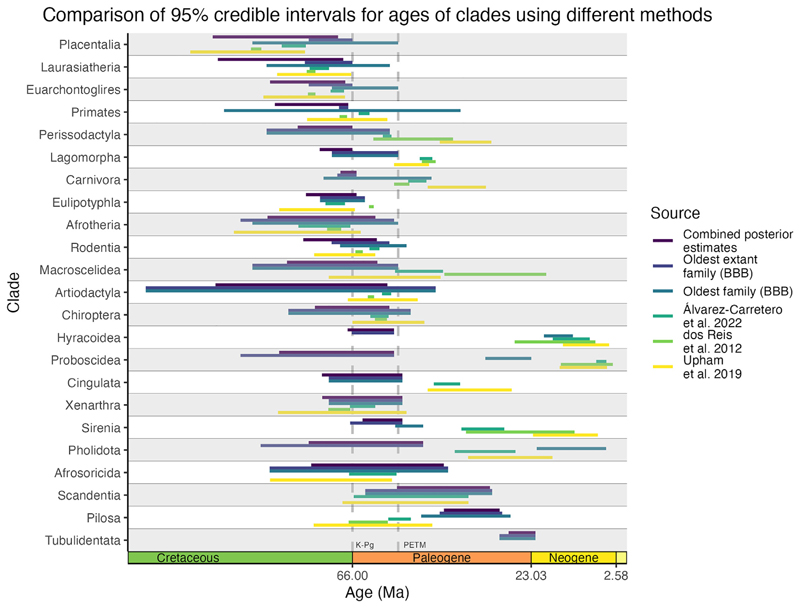
Age estimate comparisons Comparison of the combined posterior estimates for the total groups, the latest molecular clock analyses,^9–11^ the oldest family under the BBB method, and the oldest crown (typically extant) family under the BBB method. Significant overlap exists between the BBB estimates and the molecular clock analysis results for many of the clades.

Instead of running the analysis on higher taxonomic ranks, we can combine the posterior estimates of the root ages for all families within a clade to obtain another indirect estimate of the age of the clade itself, calculating the probability that at least one family originated in the Cretaceous. Of the eighteen placental mammal total group orders examined here, Primates has 100% support for a Cretaceous origin, supported by the presence of “plesiadapiform” families, such as Purgatoriidae, around the K-Pg boundary. Eulipotyphla, Lagomorpha, and Carnivora show high support for a Cretaceous origin, whereas the remaining clades have less than 50% support for a Cretaceous origin. Cingulata, Afrosoricida, Macroscelidea, Proboscidea, Rodentia, Chiroptera, Artiodactyla, Pholidota, and Perissodactyla overlap the K-Pg boundary, and Pilosa, Tubulidentata, Hyracoidea, and Sirenia likely originated after the K-Pg boundary (Figure 6). The age estimate for Scandentia is based on a single family, Tupaiidae, and suggests a post-K-Pg origin for this group. This indicates that, although Placentalia likely originated in the Late Cretaceous, the majority of orders likely originated around or after the K-Pg boundary, possibly coinciding with the Paleocene-Eocene thermal maximum (PETM)^49,50^ (Figures 5 and 6).

### Molecular clock analyses

In general, the results of the BBB analyses are consistent with those of recent molecular clock estimates, with substantially overlapping credible intervals (Figure 6). Out of 23 clades from recent molecular clock analyses (Figure 6), 12 have overlapping credible intervals compared with the total group BBB analysis, suggesting strong agreement in the age estimates. Of the 10 that do not overlap, many are estimated to be older under BBB model, which may reflect the use of total group clades rather than crown group clades. Placentalia and crown Pilosa are the only clades estimated to be younger based on the BBB results. The order Pilosa (sloths and anteaters) has a substantially younger age estimate using the BBB method compared with molecular clock estimates: the combined posterior estimates for pilosan families suggest a 44–30.7 Ma origin for the total group, whereas recent molecular clock analyses have consistently placed the origin for the crown clade at 60–50 Ma.^4,9–11^ Molecular clock analyses commonly use *Pseudoglyptodon chilensis*, interpreted as sister group to all other sloths, as the fossil calibration for Pilosa, providing a minimum age of 31.2 Ma for the crown clade.^9,51,52^ *Pseudoglyptodon chilensis* was included in our analysis in the family Pseudoglyptodontidae^53^ (however, note that the PBDB has a slightly different age range from that used by the recent molecular analysis^9^), but, despite this, the root age of the clade did not reach the age suggested by molecular clock analyses. This could be a consequence of the broad uncertainty in the fossil calibrations used to constrain the age of Pilosa and the clades that encompass it. This highlights not only the challenge of defining maximum constraints in fossil calibrations but also the possibility that the BBB model may be used as a basis for objectively establishing both minimum and maximum constraints for downstream molecular clock studies. Alternatively, it could be the result of molecular clock analyses being based on more than just fossil occurrence data, including estimated substitution rates and their heterogeneity across branches.

Although the BBB model is independent of phylogeny, it is not independent of the taxonomic affinity of the fossils used, and changes to interpretation of key fossils will impact the estimated ages of clades. However, changes to fossil interpretations are easily implemented in the BBB model and do not necessitate a complete re-analysis of all data. Rather, only the relevant families and clades need to be updated and analyzed following changes to fossil affinities. This greater flexibility lends itself well to analyzing the ever-changing mammal phylogeny.

### Implications for understanding mammal evolution

The estimates of origination and extinction inform our understanding of placental mammal evolutionary history. Since our extended BBB model allows the analysis of extinct families that are lost to molecular data, it facilitates the inference of a more complete evolutionary history than is possible with molecular clock methods. Our results find high support for a Late Cretaceous origin for placental mammals, with at least seven families definitively originating within the Cretaceous. These seven families can be placed within Placentalia, but none can be placed within ordinal crown groups. Deciphering the origination of orders is more complicated, with support for either the Long Fuse Model or the Soft Explosive Model (Figure 5).^2,3,8^ The upper boundary of the credible intervals for each root age estimate supports the Long Fuse Model, with an origin for crown orders in the Late Cretaceous (Figure 5B). However, the lower boundary of the credible intervals supports the Soft Explosive Model, with orders originating at or just after the K-Pg boundary, with a Cretaceous origin for Placentalia and a radiation around the PETM^49,50^ (Figure 5C). The Explosive Model can effectively be excluded, given the strong evidence for placental mammal families from Laurasiatheria and Euarchontoglires originating in the Cretaceous. Neither Xenarthra nor Afrotheria have families definitively originating within the Cretaceous, but the combined posterior estimates for both clades suggest an origin around the K-Pg boundary, in agreement with recent molecular clock analyses^9^ (Figure 6). The family Adapisoriculidae has been interpreted as afrotherians,^45^ which, if confirmed, would result in a definitive Cretaceous origin for Afrotheria as well. At the PETM, non-placental mammals may have experienced greater numbers of extinctions than placental mammals (Figure 5A). Out of the placental mammals, Primates and Rodentia show some increased extinctions at the PETM (Figure 3), but many orders experienced family origination at and just after the PETM.

Since the BBB model does not use molecular data or a phylogenetic framework, the model is independent of the assumptions of molecular clocks and less subject to uncertainties in mammalian phylogenetic relationships. Molecular clock analyses typically first establish the phylogenetic tree that will be used in the divergence time estimation, jointly estimate both divergence times and tree topologies,^4,10,12^ or run the analysis on several competing topologies and compare the results.^9,11^ This is not necessary when using the BBB model, which requires no phylogenetic input. The BBB model is dependent on accurate taxonomic classification, but each family is run independently of each other, which allows for changes to fossil interpretations to be implemented easily and without requiring the re-analysis of the entire dataset. Any changes to clade classification only affect how the clade ages are grouped together and summarized, and changes to fossil classification require only the re-analysis of the affected families. On the other hand, a phylogeny can provide additional useful information about clade age, and future BBB developments could aim to incorporate clade relationships in their estimates. This could be achieved with a joint analysis of different clades with age constraints established by sister-clade relationships.

Despite the lack of fossils of definitive placental mammals in the Cretaceous, our interpretation of the fossil record based on the BBB model is in accordance with molecular clock estimates showing a Cretaceous origin for the clade. Our results are also compatible with independent predictions that fossils of early placentals would be difficult to distinguish from stem placentals.^22^ The ever-growing availability of digitized fossil data, coupled with robust statistical inference of clade age and its inherent uncertainties, is instrumental to tackling the challenge of inferring the origin and evolution of the main branches of the tree of life.

## Star★Methods

Detailed methods are provided in the online version of this paper and include the following:

- KEY RESOURCES TABLE
- RESOURCE AVAILABILITY
  ○ Lead contact
  ○ Data and code availability

- EXPERIMENTAL MODEL AND SUBJECT DETAILS
- METHOD DETAILS
  ○ The Bayesian Brownian Bridge model
  ○ Testing the use of the Bayesian Brownian Bridge model with extinct families
  ○ Applying the Bayesian Brownian Bridge to interpret the mammalian fossil record

- QUANTIFICATION AND STATISTICAL ANALYSIS

## Star★Methods

### Key Resources Table

**Table:** 

REAGENT or RESOURCE	SOURCE	IDENTIFIER
Deposited data		
Fossil data	This study	Data S2
Modern species data	This study	Data S3
BBB Results	This study	Data S1
Raw fossil data	The Paleobiology Database/this study	Data S4
Output log files	This study	https://doi.org/10.6084/m9.figshare.23268524
Software and algorithms		
rootBBB model	This study	https://github.com/dsilvestro/rootBBB;https://doi.org/10.6084/m9.figshare.23268524

### Resource Availability

#### Lead contact

Further information and requests for resources should be directed to and will be fulfilled by the lead contact, Emily Carlisle (emmy.carlisle@gmail.com).

#### Data and code availability

- The results of the BBB analysis for mammal families can be found in Data S1. For each family is reported the number of extant species (N_extant), the number of fossils (N_fossils), the age of the oldest fossil (oldest_fossil) and the age of the youngest fossil (youngest_fossil). The results from the BBB model are provided as root age estimate (root_est) and lower and upper 95% credible intervals (root_lower, root_upper); extinction age estimate (ext_est) and lower and upper 95% credible intervals (ext_lower, ext_upper); the sampling rate (q_est, q_lower, q_upper); the trend parameter (a_est, a_lower, a_upper); and the Brownian bridge rate (sig2_est, sig2_lower, sig2_upper). Clade assignments for each family are listed in order of broadest to narrowest, and whether the family is stem or crown at order level is provided. We also provide the number of horizons, the stratigraphic range (strat_range), and the alpha used to estimate the stratigraphic confidence intervals for each family (marsh_origin_lower, marsh_origin_upper, marsh_ext_lower, marsh_ext_upper). Note that the number of extant species may be higher due to the inclusion of recently extinct species from the first time bin, and that the number of fossils records diversity through time not abundance. Data S2 provides the fossil occurrences through time, binned into 1-million-year time bins, used in the analysis. Modern diversity numbers for each family are provided in Data S3, and the raw data from the Paleobiology Database is in Data S4. Output files for each family from the BBB analyses can be found at https://doi.org/10.6084/m9.figshare.23268524.
- All original code implementing the BBB model has been deposited on Figshare at https://doi.org/10.6084/m9.figshare. 23268524 and is publicly available as of the date of publication.
- Any additional information required to reanalyze the data reported in this paper is available from the lead contact upon request.

### Experimental Model And Subject Details

The most recent version of the BBB model can be found at https://github.com/dsilvestro/rootBBB. The version used in this analysis can be found on Figshare at https://doi.org/10.6084/m9.figshare.23268524.

## Method Details

### The Bayesian Brownian Bridge model

The BBB model, applied to clades with living descendants, assumes that the diversity of a clade follows a random walk, modelled through a Brownian bridge, constrained at the origin (where the diversity is one) and the present-day diversity. Molecular clock models typically describe the diversification history of a clade using a birth-death process and estimating speciation and extinction rates (e.g. Zhang et al.^31^ and Wright^54^). In contrast the BBB model simulates diversity trajectories, resulting from an unknown underlying speciation and extinction process, that are compatible with both the fossil record and the present diversity of the clade. Rather than attempting to estimate the single best fitting trajectory, this framework integrates across a wide range of plausible diversification histories based on random walks, thus accounting for different scenarios such as linear or exponential diversity increase, wax and wane, and various degrees of fluctuations in species richness (see Figure 1 in Silvestro et al.^33^). The model uses two input datasets: a vector of sampled diversity of the clade through time based on the number of species known from the fossil record, and present-day species richness. The model estimates the time of origin of the clade, the variance of the Brownian bridge, describing how rapidly diversity can change through time, and the parameters modelling the sampling probability through time.

The vector of sampled diversity is assigned to pre-defined time bins, here set to 1 million years in duration to accommodate the resolution needed to examine families near the present. In our datasets, many of the bins had zero sampled species due the incompleteness of the fossil record. The model uses data augmentation to generate vectors of unknown true diversity between the estimated point of origin and the present-day diversity.^33^ This is further conditioned such that (i) the clade cannot go extinct before the present, even if there are no fossils in the intervening time bins, and (ii) the estimated true diversity cannot drop below the sampled diversity in any time bin. The model can accommodate increasing sampling rates in the fossil record towards the present^21,55^ and the resulting low sampling rates near the origin of a clade.^56^ This is achieved through the inclusion of a parameter modelling an exponential increase in sampling rate as a function of time, with the magnitude of this exponential increase estimated by the model. Although more complex models of sampling rate heterogeneity are possible, they would not be applicable to clades with scarce fossil records.^33^

Originally applied to angiosperms,^33^ the model only estimated the age of extant families, using the modern diversity as one endpoint for the Brownian bridge. Although this had little impact on the estimated age of angiosperms, for which every fossil is placed into an extant family, this would result in the loss of many extinct placental mammals that inform the early evolutionary history of the clade. Here we extend the model to the analysis of extinct clades. We add a parameter that models the unknown time of extinction with diversity constrained to zero and estimate it within the time range spanning from the most recent fossil to the present. As a result, the model jointly estimates the times of clade origination and extinction, the variance of the Brownian bridge, and the parameters quantifying the sampling rate and its temporal change. To validate the use of the extended BBB model with extinct taxa, we used simulations.

### Testing the use of the Bayesian Brownian Bridge model with extinct families

The BBB model was previously validated to ascertain how accurate its estimates of clade ages are.^33^ However, with the inclusion of extinct clades into the model, new validation tests were required. Many mammal families are extinct and some of these represent the earliest placental mammals; accurately estimating the ages of origin and extinction of these families is important for understanding mammal evolutionary history. To validate the extended model, we simulated 200 datasets of extinct families with a clade age randomly sampled from a uniform distribution *t*_*origin*_ ~ U[100, 600] Ma and a time of extinction sampled from a uniform distribution *t*_*extinction*_ ~ U[10, 0.9 * *t*_*origin*_] Ma. Analyses were run on a time-increasing rate model with different sampling rates drawn randomly for each time bin,^33^ with a mean rate of ~0.0067, i.e. about one in 150 lineages is expected to leave a fossil record in a time bin, on average. A random sample of the time bins were assigned a sampling rate of zero to simulate gaps in the fossil record. We analysed the simulated datasets through 50,000 Markov chain Monte Carlo (MCMC) iterations to obtain posterior samples of the BBB model parameters. We used the posterior mean and 95% credible intervals of the estimated clade age and time of extinction to assess the accuracy of the estimations against the true generating values, which we quantified as relative errors: (x_estimated_ – x_true_) / x_true_.

In the 200 simulated datasets, estimates of the ages of origin and extinction were unbiased under the updated BBB model (Figure 2), with relative errors centred around zero (root absolute mean of 0.023, standard deviation of 0.024; extinction absolute mean of 0.065, standard deviation of 0.103). The size of the 95% credible intervals is centred on 10-20 million years (Myr) in length, with larger intervals associated with datasets with fewer fossils, as anticipated. The log variances were slightly underestimated throughout, but the estimated times of origin remained unbiased around zero (Figure S1). The estimated sampling rates (q) ranged from 0.006 to 1.325 (median of 0.056, standard deviation of 0.146; Figure S3) and the trend parameter (a) ranged from 0.0498 to 73.205 (mean of 2.07, standard deviation of 6.495; Figure S4). The average number of time bins with zero sampled diversity was 51.31%, with a maximum of 88.24% and a minimum of 15.17% (Figure S2). Simulations were extended up to 600 Ma, long before mammals are expected to have originated, to assess the model’s accuracy for both young and old clades.

The simulation tests demonstrate that the model is accurate at estimating both the true origin and true extinction ages even when the fossil record is poor (Figure S5), and the underestimation of the true variances of the clades does not have a biasing effect on the time of origin.^33^ Given this, the model is suitable for estimating the extinction and origination age of placental mammals, many of which have poor fossil records especially at the origin of the clade.

### Applying the Bayesian Brownian Bridge to interpret the mammalian fossil record

The model was applied to an empirical dataset of 539 mammal families, of which 380 were placental mammals and the rest marsupials, monotremes and extinct basal mammals. A total of 22,381 fossils were used, of which 19,443 were placental mammal fossils (Data S2 and S3). The data were downloaded from the Paleobiology Database (PBDB) on 3 August 2022 and are available in Data S2 and S4. We conducted the analysis at family level (i.e., estimating the age of each family based on the diversity of species through time) as this represents the smallest rank that provides enough fossil data to be analysed while still allowing for different sampling rates among lineages.^33^ The data were cleaned by removing any species that did not have an assigned family, any fossil occurrences (i.e. fossils from the same collection) without an assigned species, and any fossil occurrences with an age range of greater than 20 million years, which were deemed to be too uncertain to be included. Trace fossils were also excluded from the dataset. Extensive manual checks of the fossil occurrences were performed to ensure good quality data, and records that were updated were also corrected on the PBDB. Out of the initial download of 61,100 records for all of Mammalia, 38,719 were removed primarily due to being duplicate species within the same time bin, but some were removed because of age uncertainty (age ranges greater than 20 million years) or inaccuracies in classification data. The age of each collection was randomly drawn from the age range supplied by PBDB, and each fossil species from the same collection was assigned that age. Duplicate species within the same time bin were removed. The data were binned into 1-million-year time bins and each family was analysed independently using the BBB model for 1,000,000 iterations or until it reached convergence. Modern species numbers for extant families were from Burgin et al.^57^ (Data S3). The model and output files from the analyses can be found at https://doi.org/10.6084/m9.figshare.23268524. We examined the results in Tracer v.1.7.1 to confirm convergence, quantified through an effective sampling size (ESS) greater than 100, and lineages through time plots were created using the 95% credible intervals around the times of origination and, if applicable, extinction for each family.

To compare with other interpretations of the fossil record, a confidence interval for each family was calculated based on the stratigraphic range, using equations from Marshall et al.^25^ This method uses the number of fossiliferous horizons as well as the stratigraphic range between the youngest and oldest horizons to calculate the confidence interval for the age of origin. It can also be applied to the age of extinction^24,25^ The number of fossiliferous horizons for each family was calculated based on the number of unique collections on PBDB. Confidence intervals were calculated for both root ages and extinction ages (where extinct) using the equations in Marshall et al.^25^

### Quantification And Statistical Analysis

Details of statistical analyses can be found in the results and STAR Methods Details. We assessed the convergence of the BBB model using Tracer v1.7.1 and following the methodology in Silvestro et al.^33^ Correlation coefficients for Figures S3 and S4 were calculated in R using the stat_cor() function from the package tidyverse.

## Supplementary Material

Supplemental information can be found online at https://doi.org/10.1016/j.cub.2023.06.016.

Data S1

Supplementary material

## Highlights

- The Bayesian Brownian bridge model estimates clade ages based on the fossil record
- The model shows that placental mammals originated in the Cretaceous
- Diversification of placental orders occurred after the K-Pg mass extinction
- Clade age estimates can be obtained without molecular data or phylogenetic models

## In brief

Carlisle et al. use a Bayesian model to estimate the age of the origin of placental mammals based on the stratigraphic ranges and diversity of fossil and living taxa. The results point to a Late Cretaceous origin for placental mammals, in agreement with recent molecular timescales, with ordinal crown groups originating at or after the K-Pg boundary.

## References

[1] Springer MS, Foley NM, Brady PL, Gatesy J, Murphy WJ (2019). Evolutionary models for the diversification of placental mammals across the KPg boundary. Front Genet.

[2] Murphy WJ, Foley NM, Bredemeyer KR, Gatesy J, Springer MS, Lewin HA, Roberts RM (2021). Annual Review of Animal Biosciences.

[3] Archibald JD, Deutschman DH (2001). Quantitative analysis of the timing of the origin and diversification of extant placental orders. J Mamm Evol.

[4] O’Leary MA, Bloch JI, Flynn JJ, Gaudin TJ, Giallombardo A, Giannini NP, Goldberg SL, Kraatz BP, Luo ZX, Meng J (2013). The placental mammal ancestor and the post-K-Pg radiation of placentals. Science.

[5] Halliday TJD, Upchurch P, Goswami A (2017). Resolving the relationships of Paleocene placental mammals. Biol Rev Camb Philos Soc.

[6] Cunningham JA, Liu AG, Bengtson S, Donoghue PCJ (2017). The origin of animals: can molecular clocks and the fossil record be reconciled?. BioEssays.

[7] Donoghue PCJ, Yang ZH (2016). The evolution of methods for establishing evolutionary timescales. Philos Trans R Soc Lond B Biol Sci.

[8] Phillips MJ (2016). Geomolecular dating and the origin of placental mammals. Syst Biol.

[9] Álvarez-Carretero S, Tamuri AU, Battini M, Nascimento FF, Carlisle E, Asher RJ, Yang Z, Donoghue PCJ, dos Reis M (2022). A species-level timeline of mammal evolution integrating phylogenomic data. Nature.

[10] Upham NS, Esselstyn JA, Jetz W (2019). Inferring the mammal tree: species-level sets of phylogenies for questions in ecology, evolution, and conservation. PLoS Biol.

[11] dos Reis M, Inoue J, Hasegawa M, Asher RJ, Donoghue PCJ, Yang ZH (2012). Phylogenomic datasets provide both precision and accuracy in estimating the timescale of placental mammal phylogeny. Proc Biol Sci.

[12] Bininda-Emonds ORP, Cardillo M, Jones KE, MacPhee RDE, Beck RMD, Grenyer R, Price SA, Vos RA, Gittleman JL, Purvis A (2007). The delayed rise of present-day mammals. Nature.

[13] Foley NM, Mason VC, Harris AJ, Bredemeyer KR, Damas J, Lewin HA, Eizirik E, Gatesy J, Karlsson EK, Lindblad-Toh K (2023). A genomic timescale for placental mammal evolution. Science.

[14] Foley NM, Springer MS, Teeling EC (2016). Mammal madness: is the mammal tree of life not yet resolved?. Philos Trans R Soc Lond B Biol Sci.

[15] Rannala B (2016). Conceptual issues in Bayesian divergence time estimation. Philos Trans R Soc Lond B Biol Sci.

[16] dos Reis M, Donoghue PCJ, Yang ZH (2016). Bayesian molecular clock dating of species divergences in the genomics era. Nat Rev Genet.

[17] Benton M, Donoghue P, Vinther J, Asher R, Friedman M, Near T (2015). Constraints on the timescale of animal evolutionary history. Palaeontol Electron.

[18] Marjanović D (2021). The making of calibration sausage exemplified by recalibrating the transcriptomic timetree of jawed vertebrates. Front Genet.

[19] Archibald JD (1999). Molecular dates and the mammalian radiation. Trends Ecol Evol.

[20] Yoder AD, Yang ZH (2000). Estimation of primate speciation dates using local molecular clocks. Mol Biol Evol.

[21] Marshall CR (2019). Using the fossil record to evaluate timetree time-scales. Front Genet.

[22] Halliday TJD, dos Reis M, Tamuri AU, Ferguson-Gow H, Yang ZH, Goswami A (2019). Rapid morphological evolution in placental mammals post-dates the origin of the crown group. Proc R Soc Lond B.

[23] Goswami A, Noirault E, Coombs EJ, Clavel J, Fabre AC, Halliday TJD, Churchill M, Curtis A, Watanabe A, Simmons NB (2022). Attenuated evolution of mammals through the Cenozoic. Science.

[24] Strauss D, Sadler PM (1989). Classical confidence-intervals and Bayesian probability estimates for ends of local taxon ranges. Math Geol.

[25] Marshall CR (1990). Confidence intervals on stratigraphic ranges. Paleobiology.

[26] Marshall CR (1990). The fossil record and estimating divergence times between lineages – maximum divergence times and the importance of reliable phylogenies. J Mol Evol.

[27] Foote M, Hunter JP, Janis CM, Sepkoski JJ (1999). Evolutionary and preservational constraints on origins of biologic groups: divergence times of eutherian mammals. Science.

[28] Louca S, Pennell MW (2020). Extant timetrees are consistent with a myriad of diversification histories. Nature.

[29] Beaulieu JM, O’Meara BC, Crane P, Donoghue MJ (2015). Heterogeneous rates of molecular evolution and diversification could explain the Triassic age estimate for angiosperms. Syst Biol.

[30] Luo AR, Duchêne DA, Zhang C, Zhu CD, Ho SYW (2020). A simulation-based evaluation of tip-dating under the fossilized birth-death process. Syst Biol.

[31] Zhang C, Stadler T, Klopfstein S, Heath TA, Ronquist F (2016). Total-evidence dating under the fossilized birth-death process. Syst Biol.

[32] Carruthers T, Scotland RW (2021). The implications of interrelated assumptions on estimates of divergence times and rates of diversification. Syst Biol.

[33] Silvestro D, Bacon CD, Ding WN, Zhang QY, Donoghue PCJ, Antonelli A, Xing YW (2021). Fossil data support a pre-Cretaceous origin of flowering plants. Nat Ecol Evol.

[34] Wilson Mantilla GP, Chester SGB, Clemens WA, Moore JR, Sprain CJ, Hovatter BT, Mitchell WS, Mans WW, Mundil R, Renne PR (2021). Earliest Palaeocene purgatoriids and the initial radiation of stem primates. R Soc Open Sci.

[35] Sosa LM, López García D (2018). Structural variation of the masseter muscle in Typotheria (Mammalia, Notoungulata). S Correl Geol.

[36] Cooper LN, Seiffert ER, Clementz M, Madar SI, Bajpai S, Hussain ST, Thewissen JGM (2014). Anthracobunids from the Middle Eocene of India and Pakistan are stem perissodactyls. PLoS One.

[37] Tabuce R (2018). New remains of Chambius kasserinensis from the Eocene of Tunisia and evaluation of proposed affinities for Macroscelidea (Mammalia, Afrotheria). Hist Biol.

[38] Zack SP, Rose KD, Holbrook LT, Kumar K, Rana RS, Smith T (2021). An enigmatic new ungulate-like mammal from the Early Eocene of India. Pap Palaeontol.

[39] De Bast ED, Smith T (2013). Reassessment of the small ‘arctocyonid’ *Prolatidens waudruae* from the Early Paleocene of Belgium, and its phylogenetic relationships with ungulate-like mammals. J Vertebr Paleontol.

[40] de Muizon CD, Billet G, Argot C, Ladevèze S, Goussard F (2015). Alcidedorbignya inopinata, a basal pantodont (Placentalia, Mammalia) from the Early Palaeocene of Bolivia: anatomy, phylogeny and palaeobiology. Geodiversitas.

[41] Bertrand OC, Shelley SL, Wible JR, Williamson TE, Holbrook LT, Chester SGB, Butler IB, Brusatte SL (2020). Virtual endocranial and inner ear endocasts of the Paleocene ‘condylarth’ Chriacus: new insight into the neurosensory system and evolution of early placental mammals. J Anat.

[42] Manz CL, Chester SGB, Bloch JI, Silcox MT, Sargis EJ (2015). New partial skeletons of Palaeocene Nyctitheriidae and evaluation of proposed euarchontan affinities. Biol Lett.

[43] Upham NS, Esselstyn JA, Jetz W (2021). Molecules and fossils tell distinct yet complementary stories of mammal diversification. Curr Biol.

[44] Goswami A, Prasad GVR, Upchurch P, Boyer DM, Seiffert ER, Verma O, Gheerbrant E, Flynn JJ (2011). A radiation of arboreal basal eutherian mammals beginning in the Late Cretaceous of India. Proc Natl Acad Sci USA.

[45] Seiffert ER (2010). The oldest and youngest records of afrosoricid placentals from the Fayum Depression of northern Egypt. Acta Palaeontol Polonica.

[46] Fox RC (2015). A revision of the Late Cretaceous-Paleocene eutherian mammal Cimolestes Marsh, 1889. Can J Earth Sci.

[47] Kelly TS (2014). Preliminary report on the mammals from Lane’s Little Jaw Site Quarry: a latest Cretaceous (earliest Puercan?) local fauna, Hell Creek Formation, southeastern Montana paludicola.

[48] Boyer DM, Prasad GVR, Krause DW, Godinot M, Goswami A, Verma O, Flynn JJ (2010). New postcrania of Deccanolestes from the Late Cretaceous of India and their bearing on the evolutionary and biogeographic history of euarchontan mammals. Naturwissenschaften.

[49] Bowen GJ, Maibauer BJ, Kraus MJ, Röhl U, Westerhold T, Steimke A, Gingerich PD, Wing SL, Clyde WC (2015). Two massive, rapid releases of carbon during the onset of the Palaeocene-Eocene thermal maximum. Nat Geosci.

[50] Westerhold T, Röhl U, Laskar J (2012). Time scale controversy: accurate orbital calibration of the Early Paleogene. Geochem Geophys Geosyst.

[51] McKenna MC, Wyss AR, Flynn JJ (2006). Paleogene pseudo-glyptodont xenarthrans from central Chile and Argentine Patagonia. Am Museum Nov.

[52] Varela L, Tambusso PS, McDonald HG, Fariña RA (2019). Phylogeny, macroevolutionary trends and historical biogeography of sloths: insights from a Bayesian morphological clock analysis. Syst Biol.

[53] Pant SR, Goswami A, Finarelli JA (2014). Complex body size trends in the evolution of sloths (Xenarthra: Pilosa). BMC Evol Biol.

[54] Wright DF (2017). Bayesian estimation of fossil phylogenies and the evolution of early to Middle Paleozoic crinoids (Echinodermata). J Paleontol.

[55] Holland SM (2016). The non-uniformity of fossil preservation. Philos Trans R Soc Lond B Biol Sci.

[56] Brocklehurst N, Upchurch P, Mannion PD, O’Connor J (2012). The completeness of the fossil record of Mesozoic birds: implications for early avian evolution. PLoS One.

[57] Burgin CJ, Colella JP, Kahn PL, Upham NS (2018). How many species of mammals are there?. J Mammal.

