# Supplementary material for: A timescale for placental mammal diversification based on Bayesian modeling of the fossil record
Source: Curr Biol. Author manuscript; Available in PMC 2024 Dec 9. (PMC7617171; doi:10.1016/j.cub.2023.06.016)
Supplement: Supplementary material [file EMS198351-supplement-Supplementary_material.pdf]

Current Biology, Volume *33*

Supplemental Information

A timescale for placental mammal diversification

based on Bayesian modeling of the fossil record

Emily Carlisle, Christine M. Janis, Davide Pisani, Philip C.J. Donoghue, and Daniele Silvestro

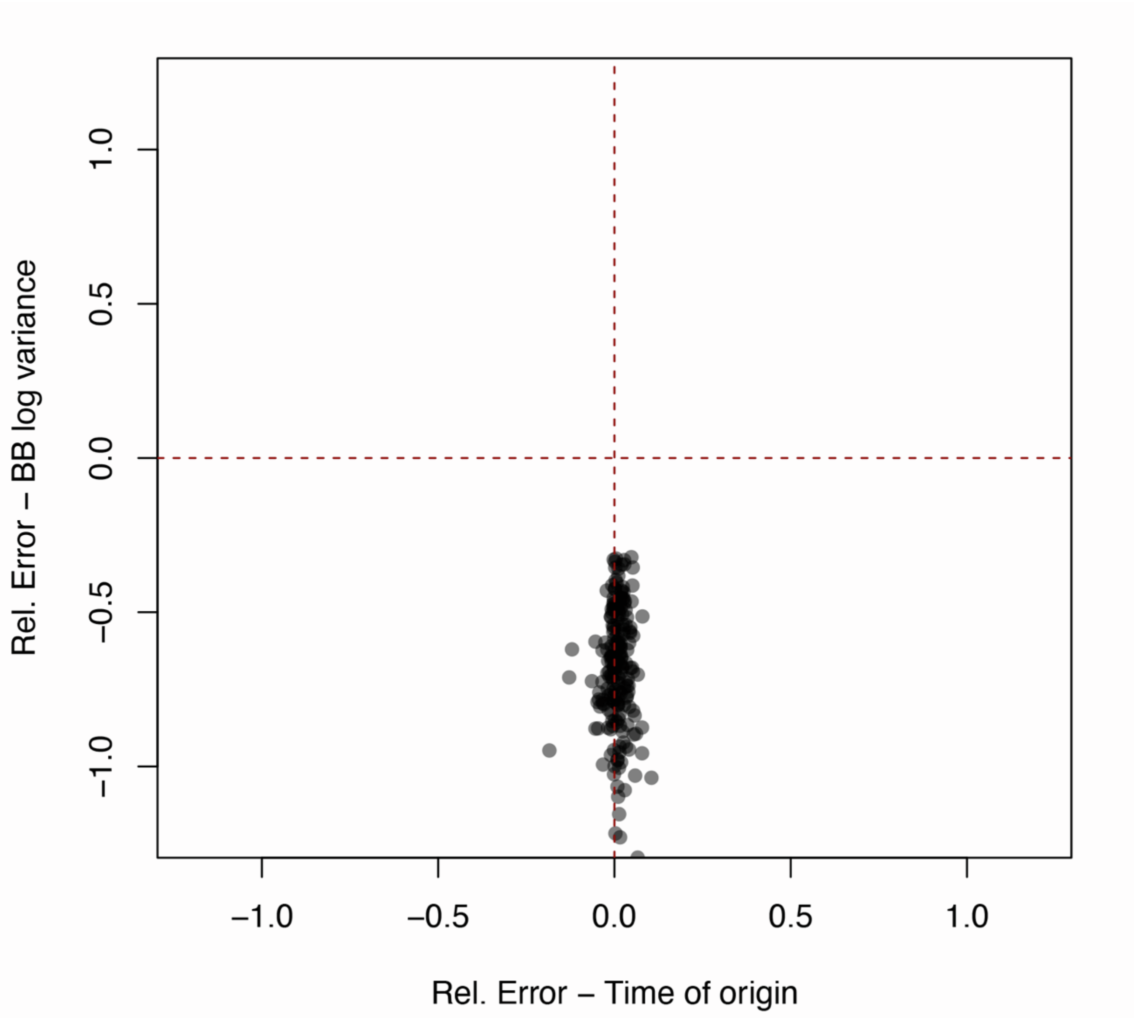

Figure S1. Relative errors of log variance and time of origin, related to STAR Methods. Although the log variance was generally underestimated, this did not have a biasing effect on the time of origin: the relative error for time of origin was still centred on zero.

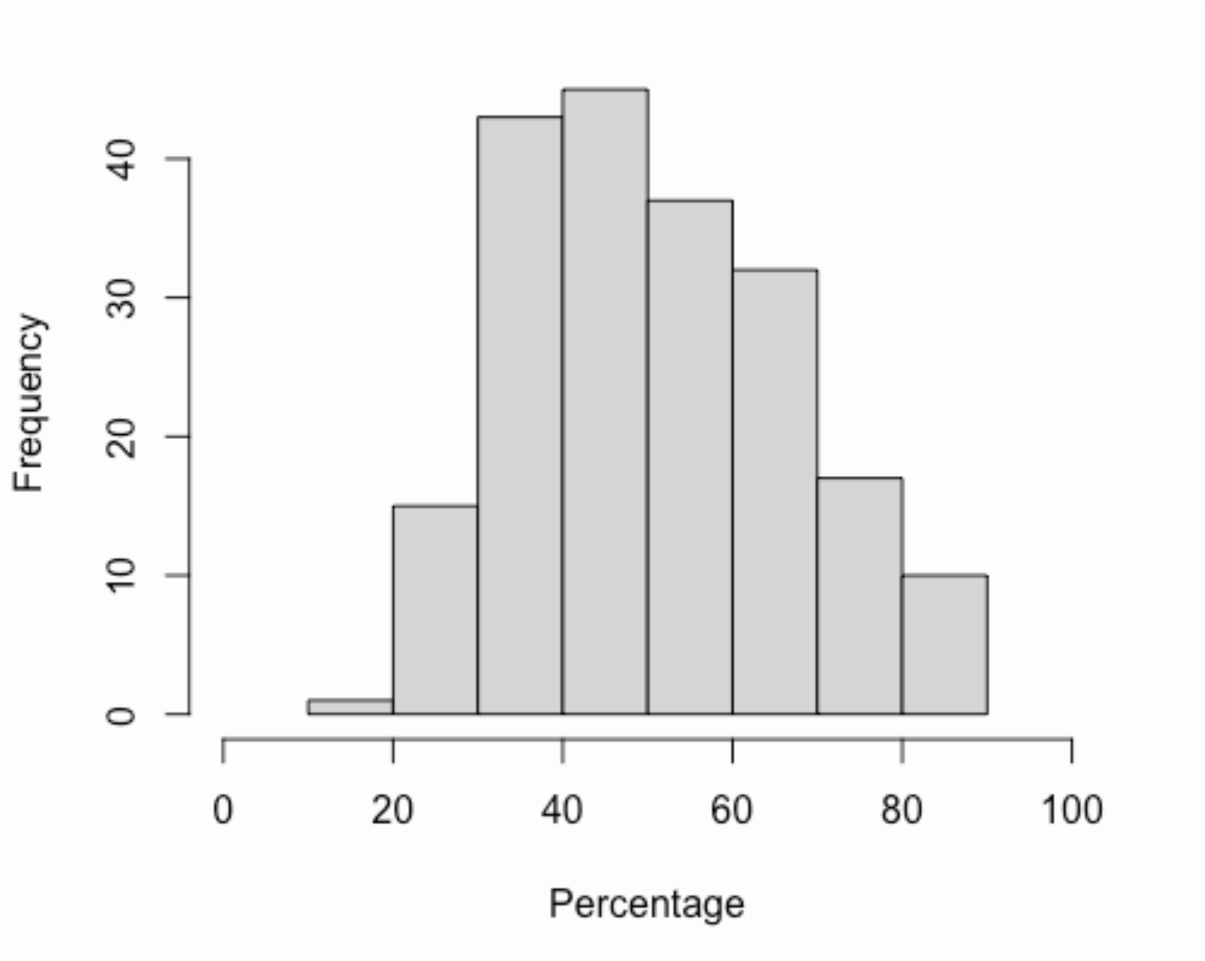

Figure S2. Unsampled time bins in simulated data, related to STAR Methods. Number of unsampled time bins (bins with zero sampled diversity) in the 200 simulation tests. Mean is 51.3%, minimum is 15.2% and maximum is 88.2%.

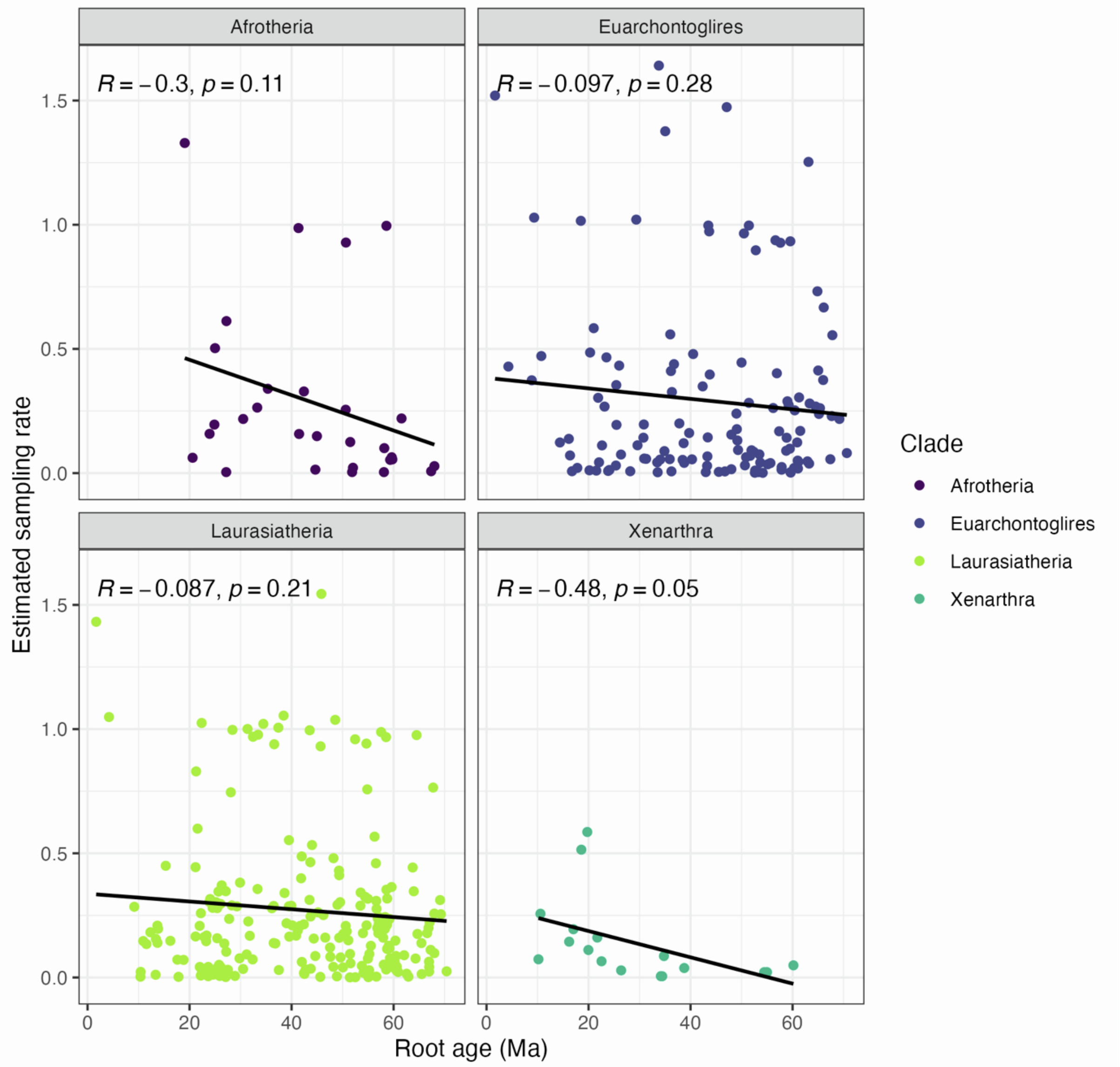

Figure S3. Root ages and estimated sampling rates in placental mammal families, related to STAR Methods. Comparison of root ages and sampling rates for placental mammal families. There are slight negative trends (i.e. as the root age of the family increases, the estimated sampling rate decreases) in all four clades.

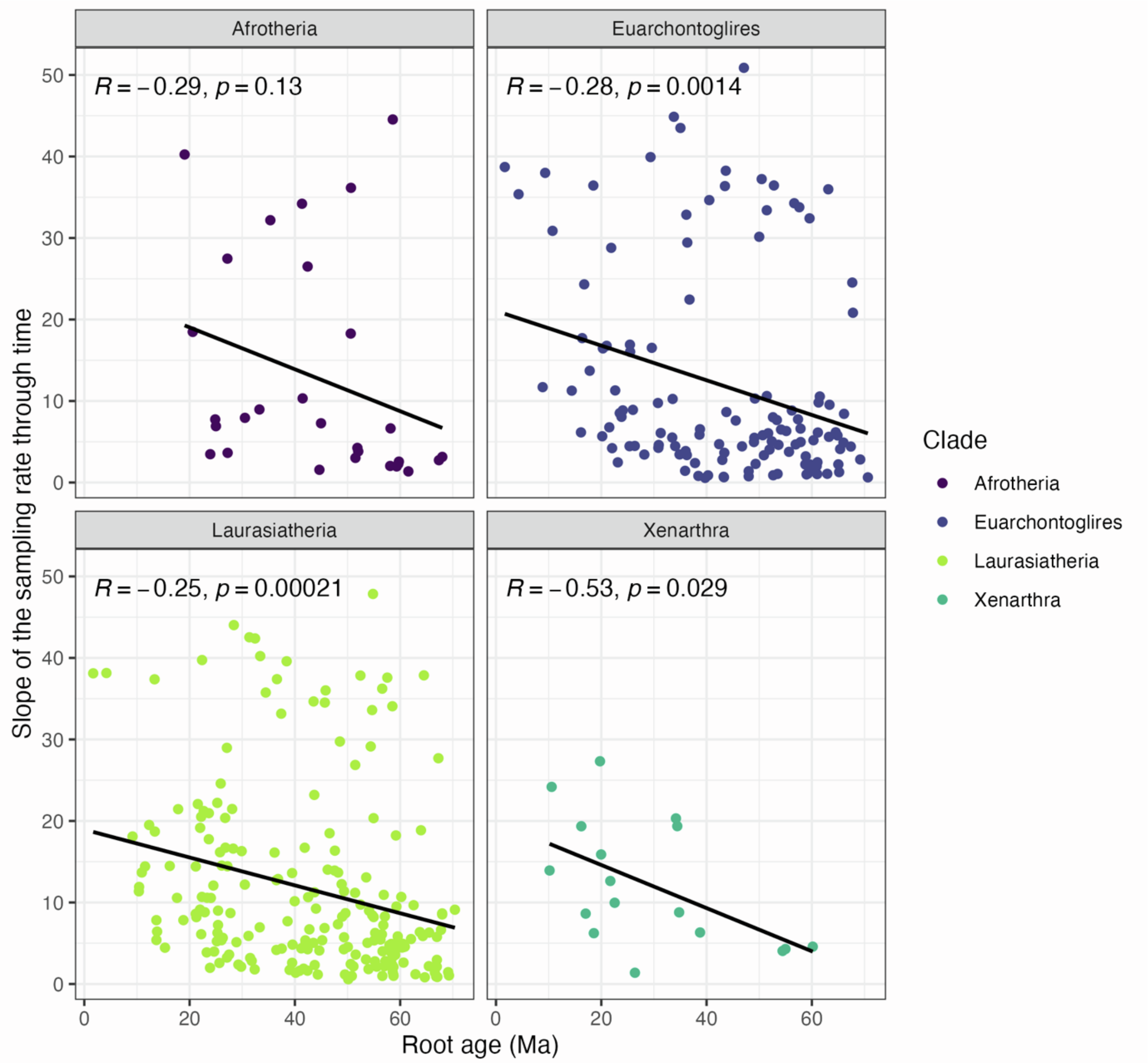

Figure S4. Root ages and the slope of the estimated sampling rates through time in placental mammal families, related to STAR Methods. Comparison of the slope of the sampling rates through time and the root ages for placental mammal families. In all four clades, as the root age increases the slope of the sampling rate through time decreases.

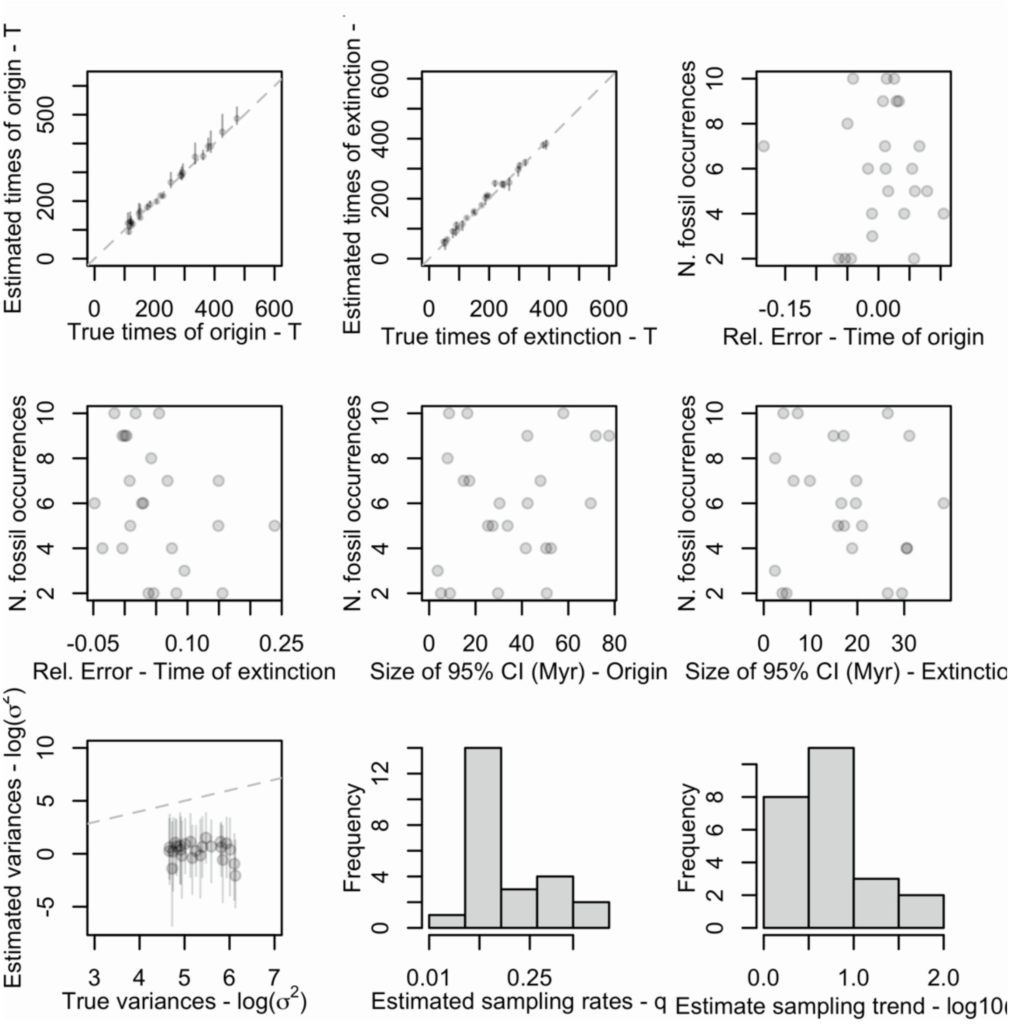

Figure S5. Simulations of 24 datasets with fewer than 10 fossils, related to STAR Methods. The time of origin and extinction are accurately estimated, with relative errors centred around zero. There is more variability in the size of the 95% credible interval, up to 80 million years for the estimate of the age of origin. The log variances were underestimated throughout, but this is seen in families with high numbers of fossils as well.
